# Mersilene tape versus conventional sutures in transvaginal cervical cerclage: a systematic review and meta-analysis

**DOI:** 10.1186/s12884-023-06141-z

**Published:** 2023-11-25

**Authors:** Juntao feng, Shisi Wei, Lihong Pang

**Affiliations:** 1 Department of Obstetrics and Gynecology, The First Affiliated Hospital of Guangxi Medical University, Shuangyong Road, Nanning, Guangxi, 530021 China; 2 Guangxi Medical University, Shuangyong Road, Nanning, Guangxi, 530021 China

**Keywords:** Mersilene tape, Suture, Cerclage, Preterm birth, Complications

## Abstract

**Objective:**

This study aimed to assess the effectiveness of Mersilene tape versus alternative suture types in prolonging singleton pregnancies as well as other pregnancy and neonatal outcomes, in cases of history-, ultrasound-, and exam-indicated cervical cerclage.

**Methods:**

A systematic review was conducted to identify relevant studies comparing different suture types in cervical cerclage procedures. The primary outcome of interest was preterm birth (PTB) rate < 37, <35, < 28, and < 24 weeks. Statistical analyses were performed to determine the relationship between suture type and various outcomes.

**Results:**

A total of five studies, including three randomized controlled trials (RCTs) and two retrospective studies, with a combined participation of 2325 individuals, were included. The pooled analysis indicated no significant association between suture type and PTB at less than 37 weeks of gestation (RR: 1.02, 95% CI: 0.65–1.60, p < 0.01, I^2^ = 74%). Women who received Mersilene tape had a higher risk of PTB at 34–37 weeks (RR: 2.62, 95% CI: 1.57–4.37, p = 0.69, I^2^ = 0%), but a lower risk of PTB at less than 34 weeks (RR: 0.43, 95% CI: 0.28–0.66, p = 0.66, I^2^ = 46%). No statistically significant differences were observed for PTB before 28 weeks (RR: 1, 95% CI: 0.65–1.53, p = 0.70, I^2^ = 0%), before 24 weeks (RR: 0.86, 95% CI: 0.60–1.23, p = 0.33, I^2^ = 0%), incidence of chorioamnionitis (RR: 0.97, 95% CI: 020-4.83, p < 0.01, I^2^ = 95%), neonatal intensive care unit (NICU) admission (RR: 0.79, 95% CI: 0.28–2.22, p = 0.08, I^2^ = 67%) and neonatal death (RR: 1.00, 95% CI: 0.42–2.35, p = 0.17, I^2^ = 48%).

**Conclusion:**

Our findings suggest that Mersilene tape does not reduce the risk of PTB before 37, 28 or 24 weeks. We observed higher risk of preterm birth between 34 and 37 weeks with Mersilene tape but lower incidence before 34 weeks, a period with higher neonatal morbidity and mortality. Due to the limited number of studies, our results and their clinical significance should be interpreted with caution.

**Supplementary Information:**

The online version contains supplementary material available at 10.1186/s12884-023-06141-z.

## Introduction

Cervical incompetence is a significant contributor to spontaneous preterm birth, characterized by the inability of the cervix to sustain a pregnancy until full term [1]. This condition can arise from either congenital factors or previous cervical damage [2].

Cerclage is a widely performed surgical procedure aimed at providing mechanical support to the cervix through the placement of suture material, thereby preventing cervical shortening and opening and reducing the risk of preterm birth(PTB) and second-trimester fetal loss [3]. While the timing and technique of cerclage have been addressed in different guidelines [4, 5], the impact of suture type on cerclage outcomes has received relatively little attention. Currently, there is no consensus on the optimal suture type for cerclage, and many surgeons may not consider suture type when performing the procedure.

Transvaginal cerclage commonly utilizes a variety of suture types, including Mersilene tape, a thick 5 mm braided polyester fiber; Ethibond, a thinner polyester thread; Prolene, a polypropylene non-braided monofilament; and Nylon [6]. Mersilene tape is the most widely used suture type for cervical cerclage due to its high strength, resistance to breakage and ease of removal. Notably, a national survey conducted in the UK among Obstetrics and Gynaecology consultants revealed a strong inclination towards using Mersilene tape [7]. However, the specific impact of Mersilene tape compared to other suture materials on pregnancy outcomes has not received adequate emphasis in current guidelines. It is crucial to recognize that the choice of suture material significantly influences surgical outcomes. In other surgical procedures, braided suture materials have been associated with an increased risk of infection [8]. Given that infection is a significant underlying factor in cerclage failure, leading to adverse outcomes such as pregnancy loss or PTB, some surgeons opt for non-braided suture materials.

To address this research gap, we conducted a meta-analysis aiming to evaluate the effects of Mersilene tape versus other suture types on cerclage outcomes. Our objective is to provide valuable insights that can inform clinical practice guidelines and recommendations for optimizing cerclage procedures in the management of cervical incompetence.

## Methods

This meta-analysis is reported in accordance with PRISMA (preferred reporting items for systematic reviews and meta analyses) guidelines. The supporting PRISMA checklist of this review is available as supporting information, see S1 Checklist.

### Search strategy

To identify eligible trials, we conducted a comprehensive search using the online databases PubMed, EMBASE, and Cochrane Central Register of Controlled Trials. The last search was performed on June 1, 2023, without any date restrictions. We employed a combination of relevant keywords and their variations, including “braided suture,“ “Mersilene tape,“ “monofilament suture,“ “suture material,“ “cerclage,“ “preterm birth” and” outcomes.” The search strategy can be found in S2 Appendix. In addition, we reviewed the reference lists of retrieved studies to identify any additional relevant articles.

### Inclusion criteria

In this systematic review, we included randomized controlled trials (RCTs), quasi-RCTs, and cohort studies that compared the use of Mersilene tape with other conventional sutures in transvaginal cervical cerclage for the prevention of preterm birth in singleton pregnancies. We included studies involving participants with history-, ultrasound- and physical examination-indicated transvaginal cerclage to prevent preterm birth, as well as any type of cerclage technique (Shirodkar, McDonald, cervicoisthmic). History-indicated cerclage encompassed placement after ≥ 1 prior mid-trimester pregnancy loss or early spontaneous preterm birth (< 28 weeks) suggestive of cervical insufficiency. Ultrasound-indicated cerclage involved placement in women with a history of prior spontaneous preterm birth and ultrasound-confirmed cervical length < 25 mm. Exam-indicated cerclage referred to placement after asymptomatic mid-trimester cervical dilation of ≥ 1 centimeter via digital examination [6]. The studies were required to report on maternal, fetal, or neonatal outcomes and provide data on the occurrence of adverse events. Only studies published in the English language were considered for inclusion in this review.

### Exclusion criteria

This review specifically aimed to compare different suture types in conventional transvaginal cervical cerclage. Therefore, other types of cerclage procedures such as abdominal, laparoscopic, and emergency cerclages, as well as replacements of cerclages, were excluded from the analysis. Additionally, twin pregnancies, ongoing trials, case reports, reviews, and animal studies were not included in the tabulation of studies. Meeting abstracts were also excluded unless we were able to obtain complete study data either from the authors or through database publications.

### Data extraction and principal analysis

Two independent reviewers (J.T.F. and S.S.W.) conducted data extraction, collecting relevant information for analysis. Any disagreements were resolved through discussion, and in cases where a consensus could not be reached, the article was excluded from the study. The extracted information included the names of the authors, year of publication, sample size, study type, indication for cerclage, cervical length at screening, gestational age at delivery, type of cerclage, and type of suture used.

The primary outcomes of interest were the incidence of PTB at gestational ages < 24, <28, < 32, <34, and < 37 weeks. Secondary outcomes, if available, included the occurrence of preterm premature rupture of membranes (PPROM), infection, chorioamnionitis, and surgical complications within 24 h of the procedure, such as hemorrhage, cervical trauma/lacerations/lesions, as well as neonatal outcomes, including neonatal mortality, fetal mortality, perinatal death, and neonatal intensive care unit (NICU) admission.

### Assessment of risk of bias

Two independent reviewers (J.T.F. and S.S.W.) conducted the critical appraisal process. Any disagreements regarding the risk of bias assessment were resolved through discussion and consensus. In cases where consensus could not be reached, a third reviewer (L.H.P.) was consulted to provide input and help resolve the disagreement.

For the assessment of RCTs, the Cochrane risk of bias tool was utilized. This tool evaluates the risk of bias in key domains such as random sequence generation, allocation concealment, blinding of participants and personnel, blinding of outcome assessment, incomplete outcome data, selective outcome reporting, and other potential sources of bias. The risk of bias for each domain was judged as either low, high, or unclear.

For cohort studies, the Newcastle-Ottawa Scale (NOS) was employed [9]. The NOS assesses the quality of non-randomized studies by evaluating three key domains: selection of study groups, comparability of groups, and assessment of outcomes. Each study is assigned a star rating based on the quality of these domains, with a higher star rating indicating a lower risk of bias. The included studies were judged as having a high (scores of 0–3), medium (scores of 4–6), or low risk of bias (scores of 7–9), respectively.

### Sensitivity analysis

A sensitivity analysis was conducted on the primary outcome. This was performed by removing studies with an overall high risk of bias to examine their impact on the effect estimate.

### Statistical analyses

Heterogeneity analysis was performed using the chi-square test, and the results were expressed as the I^2^ index. A value of 0% indicated the absence of statistical heterogeneity, while higher values indicated increased heterogeneity. A fixed-effects model was applied when heterogeneity was not significant (p > 0.1 and I^2^ < 50%). When heterogeneity was significant (p < 0.1 and I^2^ > 50%), a random-effects model was used. Pooled relative risk (RR) and corresponding 95% confidence intervals (CIs) were calculated for dichotomous variables. An RR > 1 indicated an increased risk in the intervention group compared to the control group. Publication bias was evaluated using Egger’s linear regression test, with a *p*-value < 0.05 considered statistically significant. All statistical analyses were performed using Stata 13.0 (Stata Corporation, College Station, TX) and RevMan Software Version 5.2 (Cochrane Collaboration, Copenhagen, Denmark).

## Results

### Study selection

**Fig. 1:**
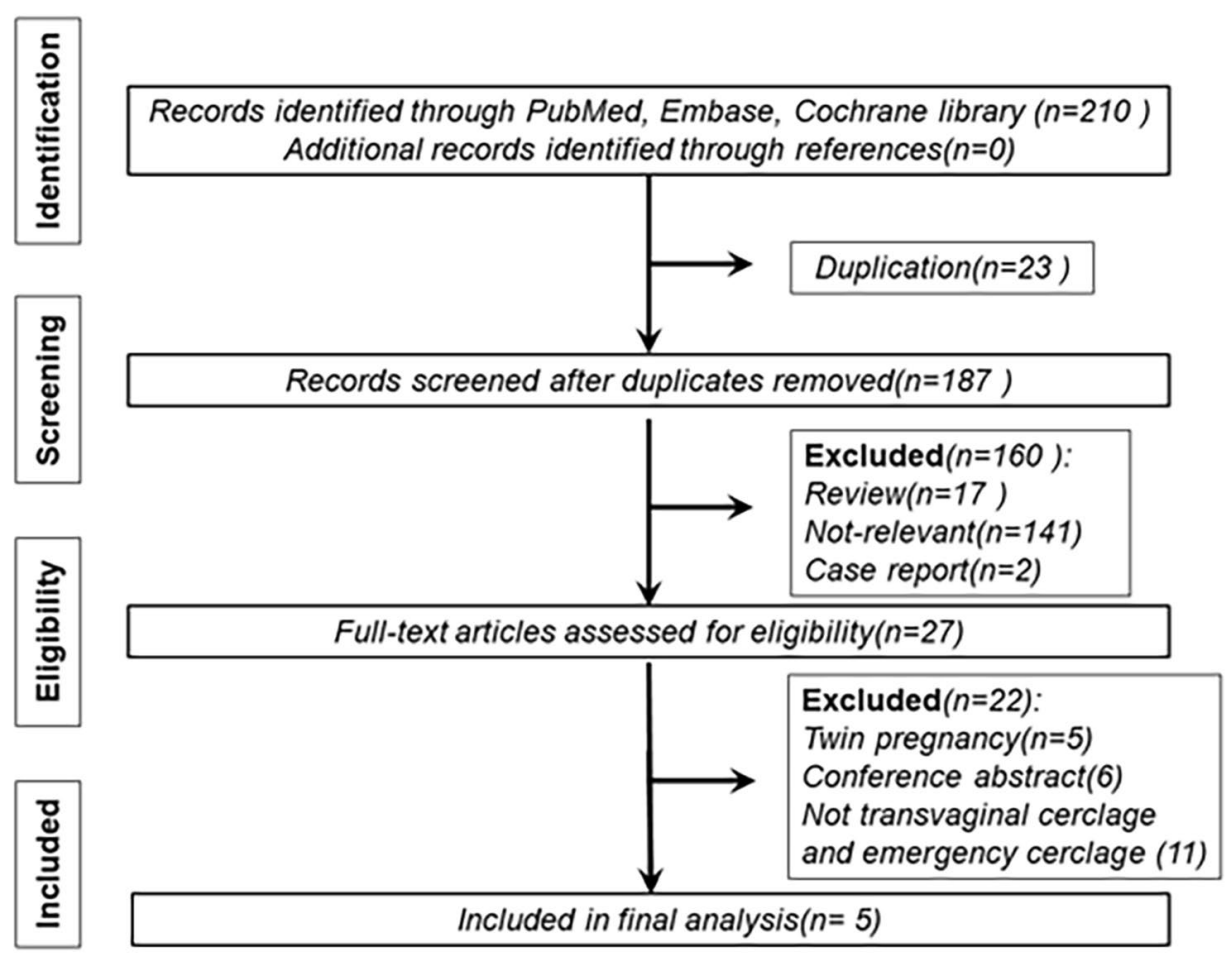
Flow diagram of the inclusion and exclusion of studies

The flowchart depicting the literature search process is presented in Fig. 1. Initially, a total of 210 studies were identified through the primary search strategy. After removing 23 duplicated articles and excluding 160 records that were either reviews, case reports, or irrelevant to the selection criteria, 27 articles underwent full-text review. Among them, 22 articles were further excluded for the following reasons: twin pregnancy (n = 5), non-transvaginal cerclage or emergency cerclage (n = 11), and insufficient or unavailable data from conference abstracts (n = 6). Ultimately, we included 5 studies [6, 10–13] that reported preterm birth rates at different gestational ages, encompassing a total of 2325 participants in our analysis.

### Study characteristics

A total of 2325 transvaginal cervical cerclages were performed to prevent preterm birth in women with indications based on history, ultrasound findings, or physical examination. Among these, 1148 cerclages utilized Mersilene tape, while the remaining 1177 cerclages employed various suture types, including Ethibond, Prolene, Ethilon, and two studies did not specify the type of monofilament used [10, 11]. Different types of cerclages were performed, including McDonald cerclage, Shirodkar, and cervicoisthmic. The studies were published between 2012 and 2022, with three conducted in the United Kingdom [10–12] and two in the USA [6, 13]. Three studies were randomized controlled trials (RCTs) [11–13], and two were retrospective cohort studies [6, 10].

Among them, the trial conducted by Morton et al. is the largest and most well-defined multicenter RCT in terms of outcome reporting [12]. The analysis of primary outcomes included 919 women in the Mersilene tape group and 926 women in the monofilament suture group. The sample sizes in the other included studies ranged from 49 to 203. All studies reported the preterm birth rate, specifically for births occurring before 37 weeks of gestation. Although the focus of all studies was on outcomes before 37 weeks, they used other different premature birth time points for comparisons, such as 34–37 weeks, less than 35 weeks, less than 28 weeks, or miscarriage (defined as delivery before 24 weeks) [12]. The main characteristics of the included studies are summarized in Table 1.

**Table 1:** Main characteristics of the eligible studies included in the meta-analysis

Study	Year	Type of study	Country	Total cerclages (Mersiline tape/other)	Main outcomes	Cervical length at screening	The gestation weeks of cerclage place	Gestational weeks at delivery (wk + d)	Cerclage indication	Type of cerclage	Type of suture
Lindsay M. Kindinger1	2016	retrosp. Cohort	UK	98(38/60)	Preterm birth rate	different length at different screening timepoint	NG	37.3 ± 3.4/38.4 ± 2.8	ultrasound-indicated	NG	Mersilen tape VS monofilament
Lindsay M. Kindinger2	2016	RCT	UK	49(25/24)	vaginal microbial dysbiosis, inflammation, and pregnancy outcomes	19 ± 4.5/18 ± 5.1	18 + 1 ± 3/17 + 6 ± 2.8	NG	ultrasound-indicated and history-indicated	NG	Mersilene tape VS monofilament
Vincenzo Berghella	2012	RCT	USA	130(46/84)	preterm birth rate	20.7/21	19.7 ± 2/19.6 ± 1.9	37.9/36.9	ultrasound-indicated	McDonald cerclage	Mersilene tape VS Mersilene thread and Ethibond
Ashley N. Battarbee	2019	retrosp. Cohort	USA	203(120/83)	gestational age at delivery. preterm birth less than 34 weeks, chorioamnionitis, neonatal intensive care unit admission and composite neonatal morbidity	16/11.5	14.3/15.9	37.2/36	ultrasound-indicated and history-indicated and physical exam	McDonald, Shirodkar, or cervicoisthmic	Mersilene tape VS Ethibond and Prolene
Victoria Hodgetts Morton	2022	RCT	UK	1845(919/926)	pregnancy loss, maternal and pregnancy outcomes	23.1/23.2	16.6/16.5	37.2/37.2	ultrasound-indicated and history-indicated	NG	Mersilene tape VS Ethilon

Preterm birth: < 37 weeks

Abbreviation: RCT, randomized controlled trial; retrosp, retrospective; NG not given

### Primary outcomes: preterm birth rate

**Fig. 2:**
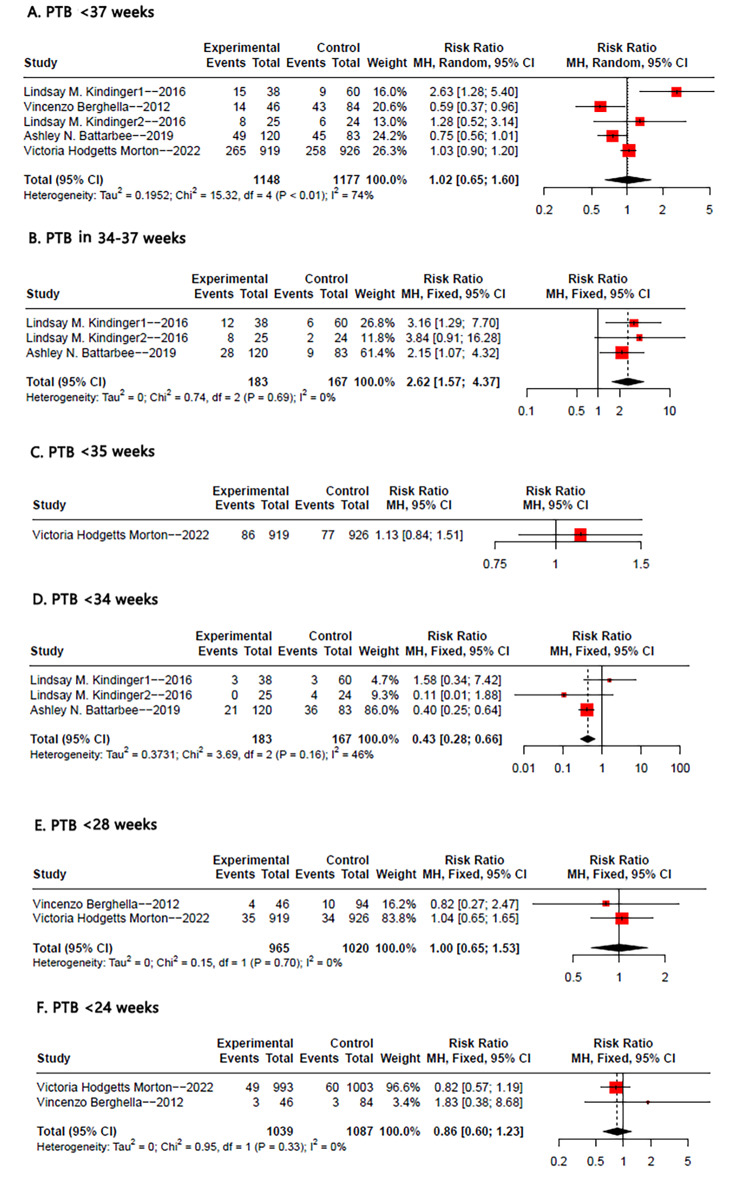
Forest plot of the pooled outcomes of (**A**) PTB<37weeks (**B**) PTB in 34-37weeks (**C**) PTB<35weeks (**D**) PTB<34weeks (**E**) PTB<28weeks (**F**) PTB<24weeks in selected studies comparing Mersilene tape to other suture types for prevention preterm birth. Abbreviations: PTB, preterm birth; CI, confidence interval; M-H, Mantel-Haenszel

Figure 2 presents the analysis for the primary outcome of preterm birth rate. All five studies were included for preterm birth rate at less than 37 weeks, and the pooled analysis revealed no statistically significant association between suture type and preterm birth (RR: 1.02, 95% CI: 0.65–1.60, p < 0.01, I^2^ = 74%). However, women who received Mersilene tape had a higher risk of preterm birth at 34–37 weeks (RR: 2.62, 95% CI: 1.57–4.37, p = 0.69, I^2^ = 0%), but a lower risk of preterm birth at less than 34 weeks (RR: 0.43, 95% CI: 0.28–0.66, p = 0.66, I^2^ = 46%). No statistically significant differences were observed for preterm birth rates at less than 28 weeks when compared to other suture types (RR: 1, 95% CI: 0.65–1.53, p = 0.70, I^2^ = 0%). Similarly, no statistically significant differences were found for preterm birth rates at less than 24 weeks (RR: 0.86, 95% CI: 0.60–1.23, p = 0.33, I^2^ = 0%). Only one study [12] analyzed the impact of suture types on the rate of preterm birth at less than 35 weeks, thus a pooled RR value could not be calculated. However, the results of this study indicated that Mersilene tape did not reduce the rate of preterm birth before 35 weeks (RR: 1.13, 95% CI: 0.84–1.51).

### Secondary outcomes

**Fig. 3:**
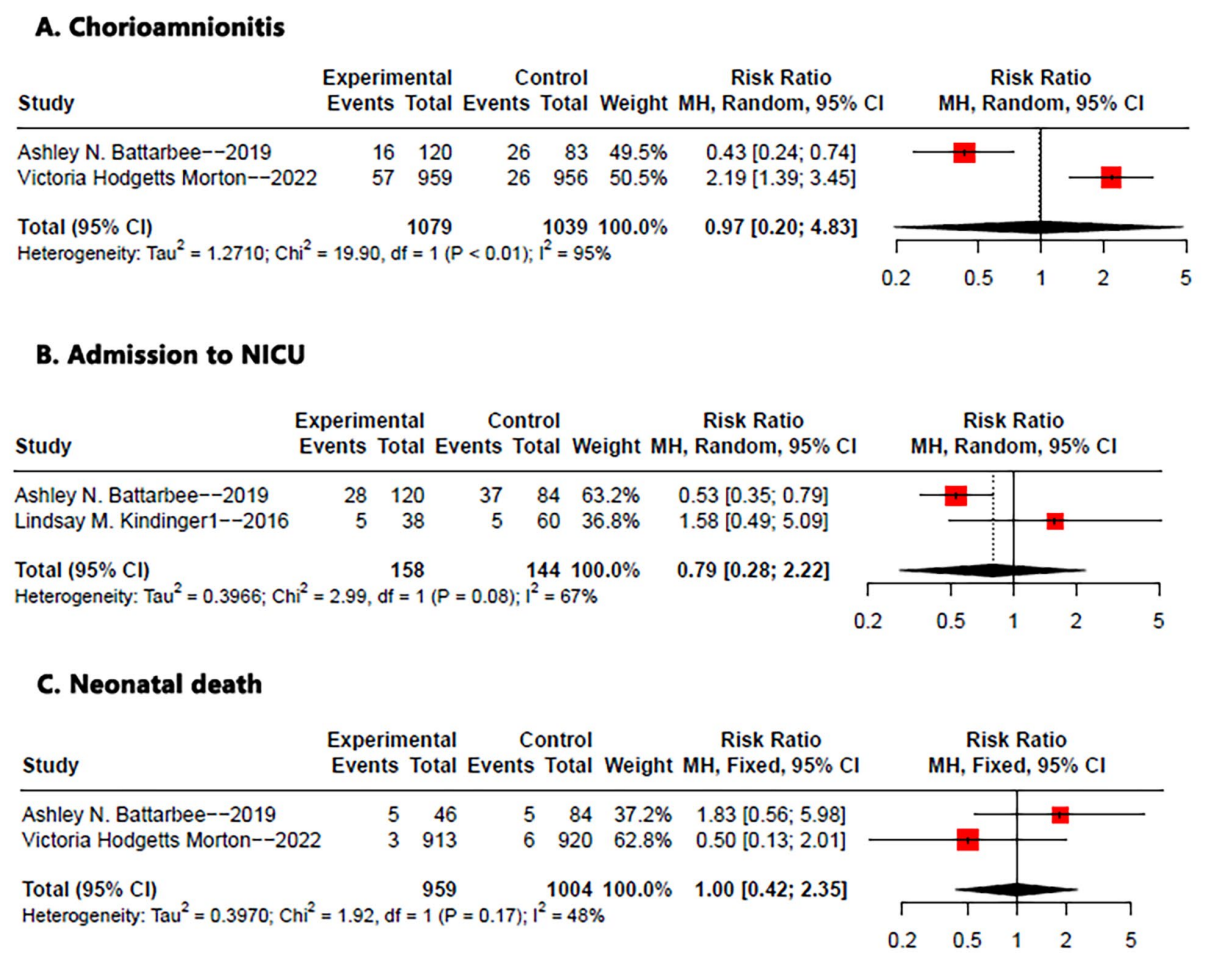
Forest plot of the pooled outcomes of (**A**) chorioamnionitis (**B**) NICU admission and (**C**) neonatal death. Abbreviations: NICU, neonatal intensive care unit; CI, confidence interval; M-H, Mantel-Haenszel

Pooled data were analyzed for three additional outcomes of interest: chorioamnionitis (RR: 0.97, 95% CI: 020-4.83, p < 0.01, I2 = 95%), neonatal intensive care unit (NICU) admission (RR: 0.79, 95% CI: 0.28–2.22, p = 0.08, I2 = 67%) and neonatal death (RR: 1.00, 95% CI: 0.42–2.35, p = 0.17, I2 = 48%) (Fig. 3). The results showed no significant difference in the use of Mersilene tape compared to other suture types for these outcomes. However, it is important to note that the evaluation of other outcomes, such as PPROM, surgical complications within 24 h of the procedure and neonatal outcomes including neonatal mortality and fetal mortality, could not be assessed as none of the included trials reported on these outcomes. Therefore, further research is needed to investigate these aspects when considering the use of Mersilene tape.

### Publication bias and sensitivity analysis

The *p*-value obtained from Egger’s test for the five included studies was 0.902, suggesting no significant publication bias. Quality assessments using the NOS indicated medium quality for the included publications (see Table S3). We assessed the risk of bias in the three included RCTs using the Cochrane Risk of Bias tool, with individual domain assessments summarized in Figure S4. In the RCT conducted by Morton et al [12], there was a lack of participant blinding, which may introduce bias in the detection of PTB. Attrition and reporting bias were also identified in the trial conducted by Berghella et al [13]. Additionally, the trial conducted by Kindinger2 et al [11] was rated as having unclear risk across all domains. Consequently, the overall risk of bias for the included RCTs is considered to be high.

**Fig. 4:**
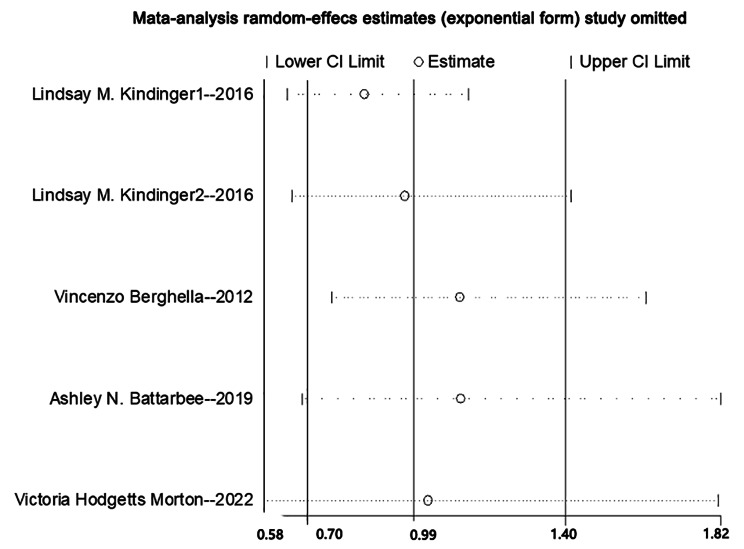
Sensitivity analysis by stepwise omission of one study at a time. The sensitivity analysis showed that no individual study significantly influenced the overall findings

The sensitivity analysis, as depicted in Fig. 4, indicated that none of the individual studies had a significant influence on the overall findings.

## Discussion

Preterm birth is a significant concern for women with cervical incompetence, and cerclage is commonly performed to prevent preterm delivery. The choice of suture type used in cerclage remains a topic of debate. Complications such as infection or suture-related trauma to the cervix can contribute to failed cerclage, and the type of suture material employed may play a role in these outcomes. The objective of our meta-analysis was to assess the impact of suture type, specifically Mersilene tape, on cerclage outcomes, particularly preterm birth rates. Our findings indicate that Mersilene tape does not confer additional benefits compared to other suture types in terms of the primary outcome of preterm birth rates at < 37 weeks, < 28 weeks and < 24 weeks. However, we did observe that the utilization of Mersilene tape seems to elevate the risk of preterm birth between 34 and 37 weeks. What’s particularly interesting, though, is that cases with Mersilene tape seem to have lower incidence of PTB < 34. This finding could have clinical significance as this group includes neonates with higher mortality and morbidity compared to those born after 34 weeks. However, it is important to note that the number of trials included in our analysis was limited, and considerable heterogeneity was observed, warranting caution in the interpretation of our results.

It appears that Mersilene tape does not offer any additional advantages compared to other suture types in terms of cerclage efficacy. The choice of suture type remains a matter of traditional practice or individual preference among obstetricians, as both Mersilene tape and other commonly used monofilament sutures have their own strengths and weaknesses. Mersilene tape, a synthetic, non-absorbable, braided polyester tape, possesses high tensile strength and excellent handling characteristics. In the Shirodkar procedure, the 5-mm-wide Mersilene tape suture is easier to visualize by ultrasound compared to the no. 2 nylon suture used in the McDonald procedure, making it easier to remove [14]. Its design is specifically engineered to provide long-term support and stability when used for suturing purposes. Additionally, due to its non-absorbable nature, Mersilene tape remains in the body indefinitely without breaking down over time. As a result, it is well-suited for applications that require long-term tissue support, such as spinal deformity correction and pelvic organ prolapse surgery [15, 16]. However, the use of this braided, non-absorbable, multifilament tape has been discontinued in urogynecology and ophthalmology due to a higher risk of complications, including erosions and infections [17, 18]. On the other hand, monofilament sutures are less flexible and may pose challenges in tying knots, with their knots being more prone to loosening due to inferior knot security [19]. Some obstetricians express concerns about the fine nature of monofilament sutures, speculating that they may cut through tissues more easily, and that removing them may require anesthesia due to difficulties in locating the threads. However, these claims lack substantial evidence in the context of cerclage and warrant further study.

Bacterial adhesion is a critical factor in the development of surgical site infections, and the physical configuration and chemical structure of suture materials play a significant role in this process [20]. A study investigating bacterial adherence at suture sites involving barbed, monofilament, and braided sutures yielded interesting findings [21]. The study revealed that the monofilament suture showed no bacterial colonization, and the monofilament portions of the barbed suture also exhibited no colonization. However, strong bacterial adherence was observed beneath the barbs of the barbed sutures. In contrast, pronounced colonies were observed in the crevices between the filaments of the braided suture. Given the more irregular surface of braided sutures compared to the smoothness of monofilament sutures, it is reasonable to speculate that, in the context of cerclage, braided sutures may serve as reservoirs for bacteria, potentially increasing the clinical infection rate or the severity of infection, ultimately leading to preterm birth. Kindinger et al. demonstrated an association between the use of Mersilene tape and shifts towards vaginal microbiome dysbiosis compared to monofilament sutures. A dynamic shift in the human vaginal microbiome towards dysbiosis has been implicated in the pathogenesis of preterm birth [11]. In line with this, the C-stitch study [12] reported no significant difference in the primary outcome between the monofilament and Mersilene tape groups, but higher rates of infection were observed with the use of Mersilene tape. However, inconsistent results were found in the research conducted by Jayakumaran et al., as they found no difference in lactobacillus prevalence among participants who had a cerclage placed using monofilament sutures compared to multifilament sutures. Additionally, no differences in maternal or neonatal outcomes were observed between the groups [22]. In this meta-analysis, when examining the rate of chorioamnionitis, the pooled results showed no significant differences between the two groups. These findings suggest that the potential of suture material to increase the risk of infection may not be the sole contributing factor to adverse outcomes such as chorioamnionitis.

In addition to the choice of suture type, it is crucial to acknowledge that other factors, such as the technique of cerclage placement, can have a significant impact on outcomes. Stirrat et al. [23] conducted a study to evaluate the cerclage technique among experienced obstetricians using a cervical cerclage simulator. The study involved 28 consultant obstetricians from 16 hospitals in 11 UK cities, who were asked to perform their “standard” cervical cerclage. The results revealed notable variations in the height and depth of suture placement and tension of the knot among these experienced obstetricians. Although these findings have yet to be confirmed in human studies, it is reasonable to speculate that such variations in cerclage technique could potentially affect the effectiveness of the procedure. Considering these observations, it becomes increasingly important to conduct further research focusing on the influence of different cerclage techniques on outcomes. By exploring the impact of various cerclage techniques, we can enhance our understanding of optimal cerclage practices and refine recommendations for clinical guidelines. These investigations should aim to provide evidence-based insights into the most effective and standardized approaches for cerclage, ultimately leading to improved outcomes for patients with cervical incompetence.

We conducted the most thorough analyses possible; nevertheless, it is crucial to recognize the limitations of this meta-analysis. Firstly, the number of available trials for inclusion was limited, which may have affected the statistical power and generalizability of our findings. Additionally, the partial reporting of data in some articles, limited to journal abstracts, may introduce publication bias. Furthermore, substantial heterogeneity was observed across the included studies, making it challenging to assess subgroup effects and potentially influenced by variations in study designs, patient populations, and clinical practices. Moreover, our study was conducted based on the older version of PRISMA checklist and therefore we did not register our protocol in advance. Another important limitation is the lack of comprehensive reporting on complications associated with different cerclage suture types. Many studies included in our analysis either lacked information or provided incomplete descriptions of surgical complications. Previous meta-analyses and case reports have reported complications such as hemorrhage, preterm premature rupture of membranes, lacerations, and bladder erosion [24]. However, the overall evidence regarding specific complications related to different suture types in cerclage procedures remains limited. Future research should focus on addressing this gap and provide detailed information on the occurrence and nature of complications.

## Conclusion

Based on the findings of our systematic review, there is currently insufficient evidence to support the superiority of Mersilene tape over other suture types in preventing preterm birth at < 37, <28, and < 24 weeks. Nevertheless, there is an observed increased risk of preterm birth (34–37 weeks) along with a reduction in preterm birth rates before 34 weeks. However, the level of evidence of the published comparative studies is limited due to the small number and the methodological heterogeneity of existing studies. This study further highlights the crucial need for more randomised controlled trials to confirm our findings and to provide more guidance for optimal use of suture types in cervical cerclage. In the absence of data suggesting the superiority of one suture type, the choice of suture for cerclage should be investigated further, and currently left to the operators’ preference.

### Electronic supplementary material

Below is the link to the electronic supplementary material.

Supplementary Material 1: S1 Checklist.

Supplementary Material 2: S2 Appendix.

Supplementary Material 3: Table S3.

Supplementary Material 4: Figure S4.

## Data Availability

All data generated or analysed during this study are included in this published article and its supplementary information files.

## Abbreviations

PRISMA: Preferred reporting items for systematic reviews and meta analyses
PTB: Preterm birth
RCTs: Randomized controlled trials
PPROM: Preterm premature rupture of membranes
CIs: Confidence intervals
NICU: Neonatal intensive care unit
RR: Relative risk
