# Supplementary material for: Mersilene tape versus conventional sutures in transvaginal cervical cerclage: a systematic review and meta-analysis
Source: BMC Pregnancy Childbirth. 2023 Nov 25;23:819. doi: 10.1186/s12884-023-06141-z (PMC10675920; doi:10.1186/s12884-023-06141-z)
Supplement: Supplementary file 2 — Supplementary Material 2: S2 Appendix. [file 12884_2023_6141_MOESM2_ESM.docx]

# S2 Appendix：Search strategy

1. Mersilene tape OR braided suture OR polyester braided thread OR monofilament suture OR non-braided thread OR Ethibond OR Prolene or Nylon OR suture material
2. cerclage OR cervical stitch
3. preterm birth OR outcomes OR efficacy
4. 1 and 2 and 3

For PubMed and Cochrane where MeSH heading are available the term “cerclage, cervical’’ [Mesh] will be added.
