# Supplementary material for: Mersilene tape versus conventional sutures in transvaginal cervical cerclage: a systematic review and meta-analysis
Source: BMC Pregnancy Childbirth. 2023 Nov 25;23:819. doi: 10.1186/s12884-023-06141-z (PMC10675920; doi:10.1186/s12884-023-06141-z)
Supplement: Supplementary file 3 — Supplementary Material 3: Table S3. [file 12884_2023_6141_MOESM3_ESM.docx]

**S3 table**: Newcastle – Ottawa quality assessment scale （cohort studies）

| **studies** | **Selection** | | | | **Comparability** | **Outcome** | | | **Score** | **Risk of bias** |
| --- | --- | --- | --- | --- | --- | --- | --- | --- | --- | --- |
|  | **Representativeness of the exposed cohort** | **Selection of the non-exposed cohort** | **Ascertainment of exposure** | **Demonstration that outcome of interest was not present at start of study** | **Comparability of cohorts on the basis of the design or analysis** | **Assessment of outcome** | **Was follow-up long enough for outcomes to occur** | **Adequacy of follow up of cohorts** |  |  |
|  | **a.** truly representative of the average (describe) in the community ☆  **b.** somewhat representative of the average in the community☆  **c.** selected group of users eg nurses, volunteers  **d.** no description of the derivation of the cohort | **a.** community controls ☆  **b.** drawn from a different source.  **c．**no description of the derivation of the non-exposed cohort | **a.** secure record (eg surgical records)☆  **b.** structured interview  **c.** written self-report.  **d.** no description | **a.**  yes (endpoint) ☆  **b.** no | a. study controls for (select the most important factor) ☆  b. study controls for any additional factor (This criteria could be modified to indicate specific control for a second important factor.) ☆ | **a.** independent blind assessment ☆  **b.** record linkage☆  **c.** self-report  **d.** no description | **a.** yes (select an adequate follow up period for outcome of interest) ☆  **b.** no | **a.** complete follow up - all subjects accounted for ☆  **b.** subjects lost to follow up unlikely to introduce bias-small number lost -> 80 % (select an adequate %) follow up, or description provided of those lost) ☆  **c.** follow up rate <_80_% (select an adequate %) and no description of those lost  **d．**no statement |  |  |
| **Lindsay M. Kindinger1** | a☆ |  | ☆ |  | a☆ | b☆ | ☆ | **a**☆ | 6 | medium |
| **Ashley N. Battarbee** | a☆ |  | ☆ |  | a☆ | b☆ | ☆ | **a**☆ | 6 | medium |

Note: A study can be awarded a maximum of one star for each numbered item within the Selection and Outcome categories. A maximum of two stars can be given for Comparability.

 Low, ≥7; medium, 5–7; high, ≤4
