# Supplementary figures and images for: Mersilene tape versus conventional sutures in transvaginal cervical cerclage: a systematic review and meta-analysis
Source: BMC Pregnancy Childbirth. 2023 Nov 25;23:819. doi: 10.1186/s12884-023-06141-z (PMC10675920; doi:10.1186/s12884-023-06141-z)

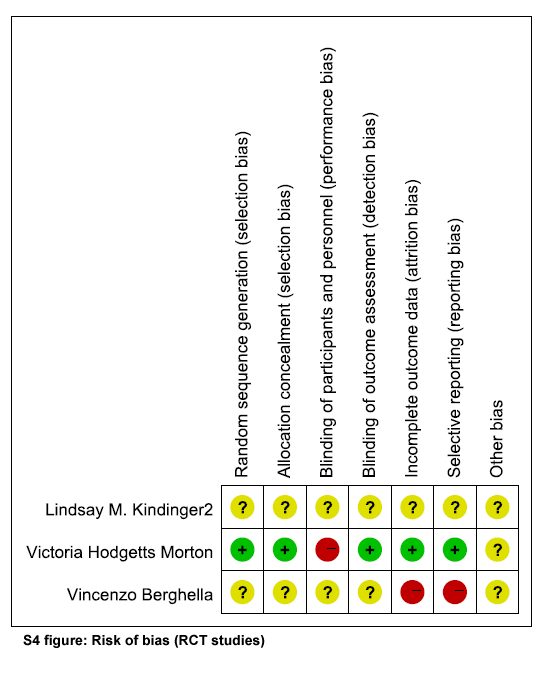

Supplement: Supplementary file 4 — Supplementary Material 4: Figure S4. [file 12884_2023_6141_MOESM4_ESM.tif]
